# Murine Susceptibility to *Leishmania amazonensis* Infection Is Influenced by Arginase-1 and Macrophages at the Lesion Site

**DOI:** 10.3389/fcimb.2021.687633

**Published:** 2021-10-01

**Authors:** Fernanda Tomiotto-Pellissier, Milena Menegazzo Miranda-Sapla, Taylon Felipe Silva, Bruna Taciane da Silva Bortoleti, Manoela Daiele Gonçalves, Virginia Márcia Concato, Ana Carolina Jacob Rodrigues, Mariana Barbosa Detoni, Larissa Staurengo-Ferrari, Waldiceu Aparecido Verri, Idessania Nazareth Costa, Carolina Panis, Ivete Conchon-Costa, Juliano Bordignon, Wander Rogério Pavanelli

**Affiliations:** ^1^Biosciences and Biotechnology Graduate Program, Carlos Chagas Institute (ICC), Fiocruz, Curitiba, Brazil; ^2^Laboratory of Immunoparasitology of Neglected Diseases and Cancer (LIDNC), Department of Pathological Sciences, State University of Londrina, Londrina, Brazil; ^3^Laboratory of Biotransformation and Phytochemistry, Department of Chemistry, State University of Londrina, Universitary Hospital, Londrina, Brazil; ^4^Laboratory of Pain, Inflammation, Neuropathy and Cancer, Department of Pathology, Center for Biological Sciences, State University of Londrina, Londrina, Brazil; ^5^Laboratory of Tumor Biology, State University of Western Parana’ (UNIOESTE), Francisco, Beltrão, Brazil; ^6^Laboratory of Molecular Virology, Carlos Chagas Institute (ICC), Fiocruz, Curitiba, Brazil

**Keywords:** Leishmaniasis, M1, M2, IFN-γ, wound healing, collagen, iNOS

## Abstract

Cutaneous leishmaniasis is a zoonotic infectious disease broadly distributed worldwide, causing a range of diseases with clinical outcomes ranging from self-healing infections to chronic disfiguring disease. The effective immune response to this infection is yet to be more comprehensively understood and is fundamental for developing drugs and vaccines. Thus, we used experimental models of susceptibility (BALB/c) and partial resistance (C57BL/6) to *Leishmania amazonensis* infection to investigate the local profile of mediators involved in the development of cutaneous leishmaniasis. We found worse disease outcome in BALB/c mice than in C57BL/6 mice, with almost 15 times higher parasitic load, ulcerated lesion formation, and higher levels of IL-6 in infected paws. In contrast, C57BL/6 presented higher levels of IFN-γ and superoxide anion ${(}^{•}O_{2}^{-})$ after 11 weeks of infection and no lesion ulcerations. A peak of local macrophages appeared after 24 h of infection in both of the studied mice strains, followed by another increase after 240 h, detected only in C57BL/6 mice. Regarding M1 and M2 macrophage phenotype markers [iNOS, MHC-II, CD206, and arginase-1 (Arg-1)], we found a pronounced increase in Arg-1 levels in BALB/c after 11 weeks of infection, whereas C57BL/6 showed an initial predomination of markers from both profiles, followed by an M2 predominance, coinciding with the second peak of macrophage infiltration, 240 h after the infection. Greater deposition of type III collagen and lesion resolution was also observed in C57BL/6 mice. The adoptive transfer of macrophages from C57BL/6 to infected BALB/c at the 11th week showed a reduction in both edema and the number of parasites at the lesion site, in addition to lower levels of Arg-1. Thus, C57BL/6 mice have a more effective response against *L. amazonensis*, based on a balance between inflammation and tissue repair, while BALB/c mice have an excessive Arg-1 production at late infection. The worst evolution seems to be influenced by recruitment of Arg-1 related macrophages, since the adoptive transfer of macrophages from C57BL/6 mice to BALB/c resulted in better outcomes, with lower levels of Arg-1.

## Introduction

Leishmaniases are a group of infectious diseases caused by species of protozoan parasites of the *Leishmania* genus, transmitted to animals and humans through the bite of female infected phlebotomine sand flies. The disease main clinical forms are cutaneous leishmaniasis (CL), visceral leishmaniasis, and mucocutaneous leishmaniasis. CL is the most common form, with estimates of 1 million new annual cases worldwide (WHO, 2020).

Classified as a neglected tropical disease by the World Health Organization, leishmaniasis represents a great challenge in the pharmacological field due to the lack of experimental vaccine candidates with satisfactory progression in human trials until now (Lage et al., 2020). Additionally, the available CL treatment is highly toxic and not fully effective (Caridha et al., 2019). A range of factors could explain the lack of vaccines and therapeutic options against CL, like the diversity among *Leishmania* species and the complexity of host’s immune responses (Müller et al., 2018; Kaye et al., 2020). Thus, understanding the effective immune response to *Leishmania* infection is fundamental to benefit the processes of drug discovery and vaccine development.

Since the discovery of T CD4^+^ helper 1 (Th1) and Th2 cells, experimental studies on CL have answered basic immunological questions related to the lesion development (Scott and Novais, 2016). Experimental studies using *L. major* have postulated that C57BL/6 mice present a predominant Th1 response associated with infection control, while BALB/c mice developed a Th2 response, favoring the disease progression (Sacks and Noben-Trauth, 2002; Scott and Novais, 2016). However, it is currently known that the Th1/Th2 paradigm is not valid for all *Leishmania* species.

In *L. amazonensis* infections, BALB/c mice also develop a lesion and a predominant Th2 response; however, C57BL/6 mice manifest a mixed immune response with mediators from both Th1 and Th2 patterns, being considered partially resistant to the infection (Soong, 2012; Pratti et al., 2016; Scott and Novais, 2016). This mixed response is similar to those observed in human infections, validating the biological relevance of these mice models for studying cutaneous leishmaniasis (Soong, 2012).

*Leishmania* parasites are known to actively manipulate their hosts and subvert the microbicide mechanisms by modifying/delaying the development of a type 1 response (Gregory and Olivier, 2005; Rossi and Fasel, 2018; Aoki et al., 2019). These escape mechanisms have been extensively characterized and vary according to the parasite species and host genetic background (Alexander et al., 1980; Lira et al., 2000; Rosas et al., 2005; Velasquez et al., 2016; Muxel et al., 2017).

Furthermore, the clinical course of *Leishmania* spp. infections does not only depend on T cells response but also involves a complex range of cells, including macrophages, the main host cells for parasite replication. Macrophages have a dual role during infection, providing a safe place for parasites’ survival inside the parasitophore vacuole, but also triggering an inflammatory response (cytokine production and oxidative stress response) that can control parasite replication. Therefore, macrophages are the key cell to disease progression, and their interaction with the parasites can dictate the success or failure of the infection (Liu and Uzonna, 2012).

There are two main macrophage types described, M1 or “classically activated” and M2 or “alternatively activated” (Mills et al., 2000). M1 macrophages are activated by the Th1 lymphocyte subpopulation, in addition to producing interferon gamma (IFN-γ) and tumor necrosis factor-alpha (TNF-α), triggering the microbicide machinery and inducing the production of reactive species, especially superoxide anion ${(}^{•}O_{2}^{-})$ by NADPH oxidase enzyme and nitric oxide (NO) by inducible nitric oxide synthase (iNOS), which eliminates *Leishmania* sp. parasites (Rossi and Fasel, 2018). However, an intense activation of M1 macrophages in the attempt to control the infection can trigger inflammation with tissue damage and exacerbation of the lesion (Laskin et al., 2011).

Conversely, M2 macrophages are activated mainly by IL-13 and IL-4 produced by Th2 cells, which, in turn, activate the enzyme arginase-1 (Arg-1), culminating in the synthesis of polyamines, allowing *Leishmania* intramacrophagic replication and favoring parasite survival and disease progression (Tomiotto-Pellissier et al., 2018). Meanwhile, despite the characteristics of permissiveness to infection, the macrophages activated by IL-4/IL-13 are also responsible for recruiting fibroblasts, keratinocytes, endothelial, and stem cells to wounds, which are fundamental to tissue repair and wound healing (Laskin et al., 2011; Krzyszczyk et al., 2018; Li et al., 2021). In CL, the resolution of skin infection is characterized by inflammation control, with lower INF-γ and TNF-α levels and higher deposition of ordered collagen fibers (Baldwin et al., 2007; Miranda et al., 2015). As iNOS and Arg-1 share L-arginine as substrate, these enzymes represent two possible pathways of immune response against *Leishmania* (Mills et al., 2000; Pessenda and Santana da Silva, 2020).

In this context, a balance between a potent microbicide response, combined with a resolutive and healing process, seems to be the key to provide the utmost benefit for the host (Tomiotto-Pellissier et al., 2018). Nonetheless, little is known about such balance and the role of M1 and M2 related molecules in leishmaniasis, whose understanding is important for developing drugs and vaccines. Based on this, our goal was to demonstrate the differential response established by mice susceptible (BALB/c) and partially resistant (C57BL/6) to *Leishmania amazonensis* infection.

## Materials and Methods

### Culture of *Leishmania (L.) amazonensis*

Promastigote forms of *Leishmania (L.) amazonensis* (MHOM/BR/1989/166MJO) and *Leishmania (L.) amazonensis* expressing an enhanced green fluorescent protein (eGFP) addressed to glycosomes (MHOM/BR/1973/M2269) were maintained in culture medium 199 (GIBCO, Invitrogen, USA) supplemented with 10% fetal bovine serum (FBS, GIBCO Invitrogen), 1 M HEPES, 0.1% human urine, 0.1% L-glutamine, 10 µg/ml penicillin and streptomycin (GIBCO Invitrogen), and 10% sodium bicarbonate (CAQ, Brazil). The eGFP strain was additionally cultured with 10 µM of geneticin G418 (Sigma Aldrich, USA) for parasite selection maintenance. The cell cultures were maintained in an incubator-type B.O.D. at 25°C in 25-cm^2^ flasks. In all experiments, promastigote forms were used in the stationary growth phase.

### Animals and Infection

BALB/c and C57BL/6 mice weighing approximately 25–30 g and aged 6–12 weeks were kept under sterile conditions and used according to protocols approved by the Institutional Animal Care and Committee. This study was approved by the Ethics Committee for Animal Experimentation of the State University of Londrina, number 8286.2016.60.

Mice were infected on the footpad of both hind paws, subcutaneously, with 10^5^ promastigote forms of *L. amazonensis/*paw. The animals were divided into groups and evaluated at 6, 24, 72, 144, and 240 h, and 11 weeks post infection (p.i.). Five to six animals were evaluated in each group/time point.

Paw edema was measured weekly using a digital caliper (Starrett 799). At each time point, both paws were measured, and the data were plotted as the mean between the right and left paws of each animal. Final data are expressed as the mean edema of animals in the group.

After the end of each time point, the animals (n ≥ 5) were euthanized by intraperitoneal inoculation of 100 mg/kg ketamine (Ceva, Brazil) and 10 mg/kg xylazine (JA, Brazil) followed by cervical dislocation, and the footpads of infected paws were collected, weighed, and processed for the next assays.

### Parasite Burden Analysis

Real-time quantitative PCR (RT-qPCR) was performed to determine the tissue parasite load in each group. Briefly, hind paw samples were mechanically homogenized (Tissue-tearor, BioSpec) in TELT buffer (50 mM de Tris-HCl pH 8, EDTA 62,5 mM, Triton-X 4% e LiCl_2_ 2.5 M), and DNA extraction was performed with the Easy-DNA kit (Invitrogen, USA, K1800-01) according to the manufacturer’s instructions. Afterward, a solution of phenol:chloroform:isoamyl alcohol (25:24:1) was added, two volumes of cold ethanol (Merck, Germany) were added to the aqueous phase, and samples were stored at -20°C for 12 h. Samples were then centrifuged for 30 min at 10,000 *g*, washed with 70% ethanol, dried at room temperature, and resuspended in 10 mM Tris-HCl (pH 8.5). Real-time PCR was performed by using GoTaq qPCR Master Mix (Promega, USA) with 100 ng total genomic DNA (gDNA). Parasite quantification was performed using AAP3 gene primer 5’-GGCGGCGGTATTATCTCGAT-3’ (Forward) 5’-ACCACGAGGTAGATGACAGACA-3’ (Reverse) *Leishmania*-specific primers at 10 pM (Tellevik et al., 2014) in a 10-µl final volume reaction. The samples were amplified with a StepOnePlus Real-Time PCR System (Applied Biosystems, USA) under the following PCR conditions steps: 2 min at 50°C, 2 min at 95°C, 40 cycles of 15 s at 95°C, 1 min at 55°C, followed by a dissociation step of 55–99°C (heating of 0.5°C/s). The results were based on a standard curve constructed with DNA from culture samples of *L. amazonensis* promastigote forms.

### Cytokines Determination

Th1, Th2, and Th17 cytokines were evaluated in the supernatant of the paw homogenates of non-infected and 11 weeks infected mice (n=5). The paws fragments were weighted, mechanically homogenized (Tissue-tearor, BioSpec) in PBS (100 mg/ml), and centrifuged (21293 *x* g, 2 min, 4°C). The supernatants were stored at -80°C, and the analyses were performed using Cytometric Beads Array (CBA) assays using a commercial kit (BD Bioscience, USA) following the manufacturer’s recommendations. The concentration of each cytokine was determined with reference to the standard curve generated from reading the different dilutions of the recombinant cytokine. The limit of detection for each cytokine was 0.1, 0.03, 1.4, 0.5, 0.9, 0.8, and 16.8 pg/ml, respectively, for IL-2, IL-4, IL-6, IFN-γ, TNF-α, IL-17, and IL-10. The data were obtained in a BD Accuri C6 flow cytometer and analyzed using the FCAP Array v. 3.0.1 software.

### Superoxide Anion Measurement

The superoxide anion ${(}^{•}O_{2}^{-})$ production in the paw homogenates was evaluated by testing with nitroblue tetrazolium (NBT, Sigma-Aldrich, USA). For the assay, one of the hind paw fragments was weighted and mechanically homogenized (Tissue-tearor, BioSpec) in KCl 1.15% and centrifuged (21293 *x* g, 2 min, 4°C). Then, 10 μl of supernatants (100 mg/ml) was plated, and 100 μl of the solution containing NBT (1 mg/ml) was added. After 15 min of reaction at room temperature, the samples were fixed with 10 μl of methanol, and the formazan, product of the reaction between NBT and ${}^{•}O_{2}^{-}$ , was solubilized by the addition of 120 μl of 2M KOH (Merck). The readings were performed on a spectrophotometer (GloMax Explorer instrument, Promega) at 660 nm, and the results normalized per milligram of tissue. Six animals were evaluated in each group/time point, and uninfected BALB/c and C57BL/6 mice were used as controls (0h).

### Determination of N-Acetylglucosaminidase Activity

For the indirect quantification of the presence of macrophages at the infection site, the activity of the enzyme N-acetylglucosaminidase (NAG) was measured. The evaluation of the cellular infiltrate in the animals’ footpad samples was performed by a colorimetric method described by Bradley et al. (1982). Briefly, the paw samples were weighed and added to 50 mM potassium phosphate buffer (pH 6.0) containing 13.72 mM HTAB (hexadecyl trimethyl ammonium bromide) and stored at -20°C. The samples (100 mg/ml) were then homogenized and centrifuged (21,293 *x* g, 2 min, 4°C). The supernatant was used for colorimetric reaction in a 96-well plate. Each aliquot of 15 μl of the sample was added to 200 μl of the reaction solution containing 52.64 mM of N-acetylglucosaminidase. The readings were performed on a spectrophotometer (GloMax Explorer instrument, Promega) at 450 nm, and the number of macrophages per milligram of tissue was determined using a standard curve.

### Histological Processing

For the immunohistochemistry, collagen quantification, and immunofluorescence, one of the paws of each animal was fixed in 10% formalin solution and after 24 h they were decalcified for 21 days in 5% nitric acid (Anidrol, Brazil) and then processed for inclusion in paraffin. The cuts (5 µm thick) were adhered to slides treated with poly-L-lysine (Sigma-Aldrich) and deparaffinized xylene (FMAIA, Brazil) and subsequently hydrated with ethanol gradients to water.

### Immunohistochemistry of Lesions for iNOS, MHC-II, Arg1, and CD206 Labeling

Typical markers of the M1 (MHC-II, iNOS) and M2 (CD206 and Arg-1) phenotypes were investigated in the histological sections of the infected mice paws. The hydrated histological sections were submitted to antigenic recovery in citrate and acetate buffer solution (pH 6) for 10 min in a microwave (40 W, 90°C). Endogenous peroxidase was blocked in a solution of 3% H_2_O_2_ (Anidrol), 10% methanol (Merck), and 0.1% Tween-80 (Sigma-Aldrich) for 30 min and, subsequently, the nonspecific sites blocked with PBS containing 1% FBS (GIBCO Invitrogen) for 30 min at room temperature. Then, the primary antibodies were added to the labeling of iNOS (NOS2, dilution 1:500, Santa Cruz Biotechnology, USA, Cat. SC7271), MHC-II (1:500, Santa Cruz, SC59318), arginase-1 (Arg-1, 1:600, Santa Cruz, SC18351), and CD206 (1:400, Santa Cruz, SC34577) for 2 h at 37°C. Subsequently, the universal solution of secondary antibodies with biotinylated antirabbit, antimouse, and antigoat IgG (LSAB + System-HRP, DAKO, Japan) was added for 1 h at room temperature. After three washes with PBS, the slides containing the samples were incubated with the avidin-peroxidase (LSAB + System-HRP, DAKO, K069011-2) complex for 40 min at room temperature. Finally, 3,3’-diaminobenzidine diaminobenzidine (DAB, DAKO, K3468) was added, and the counterstaining was performed with hematoxylin (Merck).

For determining a quantitative scoring, images were evaluated by using the color deconvolution tool from the Image J software (NIH, USA). Data are expressed in pixels (% of labeling in the image area). Five images of two sections of each animal were considered for analysis. Five to six animals were evaluated in each group/time point.

### Collagen Quantification

The quantification of collagen in the paws lesion was performed by the Sirius-red staining method, assessed under polarized light through a photomicroscope (CARL ZEISS- Axio Imager A1) with a camera (HBO 100) coupled to a computer using the AxioVision software, with a 200x magnification. Five images of two sections of each animal were considered for analysis using the Image Pro Plus software (version 4.5). The results were expressed as a percentage of area with the presence of total collagen and type III collagen in measured area (Miranda et al., 2015).

### *In Vitro* Infection of Peritoneal Macrophages

Peritoneal macrophages (1×10^5^ cells/ml) were recovered from the peritoneal cavity of non-infected BALB/c and C57BL/6 mice with cold PBS supplemented with 3% of FBS (GIBCO Invitrogen) and then cultured in 24-well plates with 200 μl of RPMI 1640 medium (10% FBS) for 2 h (37°C, 5% CO_2_). The adherent cells were infected with *L. amazonensis* eGFP promastigotes (2×10^6^ cells/ml) for 4 h for the phagocytosis of the parasites. Afterward, the non-internalized parasites were washed with PBS, and the infected cells were cultivated for additional 24 h with RPMI 1640 in the presence or absence of the arginase inhibitor N(omega)-Nitro-L-arginine methyl ester (L-NAME, Sigma) at 70 μM.

After this, the cells were stained with antibody F4/80-PE (dilution 1:100, Santa Cruz Biotech, SC377009), and the antibody staining profile and eGFP fluorescence were analyzed by flow cytometry (BD Accuri C6 flow cytometer) using the FCAP Array v3.0.1 software. A total of 30.000 events were recorded. Infection analyzes were performed considering the percentage of eGFP-positive cells, and the infection intensity was determined based on the eGFP fluorescence intensity. Three independent experiments were performed in duplicate.

### Adoptive Transfer of Macrophages

BALB/c and C57BL/6 mice were kept under the same conditions described in the section *Animals and Infection*.

Macrophages were recovered from the peritoneal cavity of non-infected C57BL/6 with cold PBS supplemented with 3% of FBS (GIBCO Invitrogen) and stained with antibodies F4/80-PE (dilution 1:100, Santa Cruz Biotech, SC377009) and CD11b-FITC (dilution 1:100, Santa Cruz Biotech, SC23937). The antibody staining profile was analyzed by flow cytometry (BD Accuri C6 flow cytometer) using the FCAP Array v3.0.1 software.

BALB/c mice were infected on both hind paws, subcutaneously, with 10^5^ promastigote forms of *L. amazonensis*/paw, and randomly divided into three groups: BALB/c adoptive transfer of macrophages (ATM) IP—animals that received the ATM with macrophages from C57BL/6 *via* intraperitoneal injection; BALB/c ATM IV—animals that received the ATM with macrophages from C57BL/6 *via* intravenous injection; and BALB/c—animals that did not receive ATM (n = 5/group).

At the ninth week of infection, the BALB/c ATM groups received the transference of 5x10^5^ peritoneal macrophages from C57BL/6 mice *via* intraperitoneal (BALB/c ATM IP) or by retro-orbital injection (BALB/c ATM IV; under anesthesia with 100 mg/kg ketamine (Ceva) and 10 mg/kg xylazine (JA) (**Figure 6A**). At the end of the 11th week, the animals were euthanized as described in the section *Animals and Infection*. The edema was measured weekly as described in the section *Animals and Infection*, the parasite burden analysis was performed as described in the section *Parasite Burden* Analysis, and the cytokines measurement was performed in the section *Cytokines Determination*.

### Enzyme-Linked Immunosorbent Assay for iNOS and Arg-1 in Paws Homogenate

Paws fragments of animal groups (n=5/group) described in the section *In vitro Infection of Peritoneal Macrophages* were processed as follows: RIPA lysis buffer (140 mM sodium chloride, 1% Triton X-100, 0.1% sodium deoxycholate, 0.5% sodium dodecyl sulfate, 1 mM EDTA, 10 mM Tris, pH 8.0, and 1 mM phenylmethanesulfonyl fluoride (PMSF)) was added (1 ml) to the samples, mechanically homogenized (Tissue-tearor, BioSpec), and incubated for 2 h, 4°C. The lysates were collected and centrifuged at 13,000 x *g* for 20 min at 4°C, the supernatants were transferred to a new tube, and the total protein concentration was quantified in NanoVue Plus GE Healthcare (Biochrom, USA). The protein concentration of all samples was normalized to 10 µg/ml, and a 50-µl aliquot was added in a 96-well ELISA plate for adsorption of proteins overnight at 4°C, followed by incubation for 1 h with blocking buffer (ELISA/ELISPOT, eBioscience, USA). The wells were washed three times with wash buffer (PBS + 0.5% Tween 20 (Sigma-Aldrich)) and incubated with primary antibody antimouse iNOS (Santa Cruz Biotech, SC7271) and Arg-1 (Santa Cruz Biotech, SC18351) at 1:500 dilutions for 1 h at room temperature. The wells were washed to remove unbound antibodies, followed by the addition of universal biotinylated secondary antibody (LSAB2 System-HRP, Dako), 1-h incubation, washing, and addition of streptavidin-HRP (LSAB2 System-HRP, Dako) for 1 h. After the incubation time, the wells were washed five times, and 100 µl of TMB Substrate Solution (eBioscience, 00-4201-56) was added, followed by incubation for 30 min, and 100 µl of stop solution (1N sulfuric acid) was added. The plate reading was performed in a microplate reader at 450 nm (GloMax, Promega), and the results are expressed as arbitrary units.

### Arginase-1 Immunofluorescence Study

The tissue sections were heated into citrate buffer 1 mM, pH 6.0, for 15 min at 100°C for antigen retrieval. Then, a working solution of bovine fetal serum 5% and Triton X 0.3% was added and incubated at 37°C in a humidified box for 15 min to block nonspecific antigens. Sections were then incubated overnight at 4°C with primary Arg-1 IgG antibody (1:500, Santa Cruz, SC18351). Afterward, they were rinsed five times in saline 0.9% for 5 min protected from light. Then, they were incubated at 37°C for 60 min with a secondary antibody (1:1,000, Alexa Fluor 488 goat anti-mouse IgG, Thermo Fischer Scientific). Subsequently, sections were rinsed two times in saline 0.9% for 5 min each. Afterward, the sections were incubated with 4,6 diamidinofenilindol (DAPI, Sigma Aldrich), 5 mg/ml solution, for 30 min. Slides were mounted with glycerol and stored protected from light for later observation under a fluorescence microscope. Representative images of five animals per group are shown.

### Statistical Analysis

Data were expressed as a mean ± SEM. Data were analyzed using the GraphPad Prism statistical software (GraphPad Software, Inc., USA, 500.288). Differences between the two groups were evaluated using the Student’s t-test. Comparisons between multiple groups were done using ANOVA followed by Tukey’s test. Differences were considered as statistically significant upon p < 0.05. At least five animals per group were evaluated.

## Results

### BALB/c Mice Show Worse Evolution of *L. amazonensis* Infection Comparing With C57BL/6 Mice

**Figure 1:**
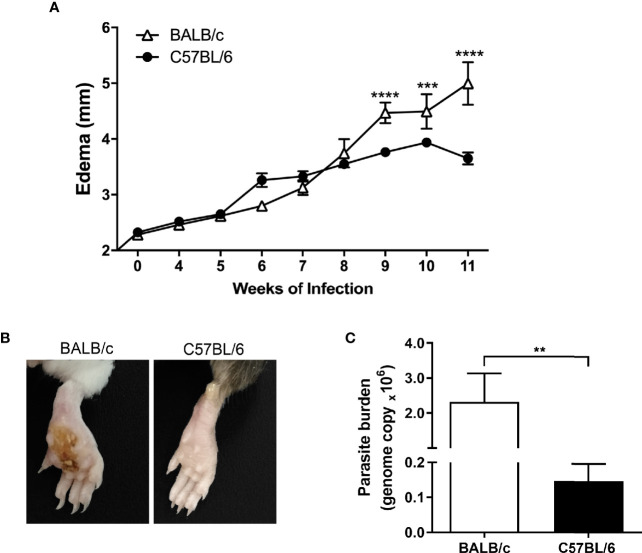
Evolution of *L. amazonensis* infection in BALB/c and C57BL/6 mice. **(A)** BALB/c and C57BL/6 mice were infected on the hind paws with 10^5^ promastigote forms of *L. amazonensis*/paw, and the edema was analyzed for 11 weeks. **(B)** Representative images of BALB/c and C57BL/6 mice paws 11 weeks after the infection with *L. amazonensis*. **(C)** Parasitic load (number of *Leishmania amazonensis* kDNA copies) determined in the mouse paw homogenate by quantitative real-time PCR after 11 weeks of infection. Data represent the mean ± SEM of six mice groups. **Significant difference in relation to the opposite strain infected by *L. amazonensis*, p ≤ 0.01, ***p ≤ 0.001, ****p ≤ 0.0001.

We analyzed the evolution of *L. amazonensis* infection through the paw lesion edema, measured weekly until the 11th week after infection. The edema was similar on both BALB/c and C57BL/6 mice until the eighth week of infection; however, from the ninth week on, the BALB/c mice showed significantly higher edema than C57BL/6 (**Figure 1A**). Also, all BALB/c mice showed ulcerated lesions after 11 weeks of infection, while none of the C57BL/6 strain had ulcerations (**Figure 1B**).

Regarding the parasitic load, we found significantly more parasites in the infected paws of BALB/c mice than in the C57BL/6 mice after 11 weeks of infection (**Figure 1C**).

### Cytokines Are Differentially Produced by *L. amazonensis*-Infected BALB/c and C57BL/6 Mice Paw

**Figure 2:**
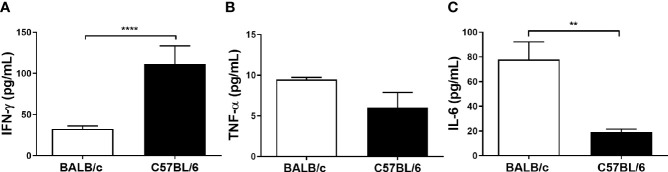
Cytokine levels of BALB/c and C57BL/6 mice infected with *L. amazonensis*. Homogenates of *L. amazonensis*-infected BALB/c and C57BL/6 paws for 11 weeks were submitted to the CBA assay. **(A)** Measurement of IFN-γ, **(B)** TNF-α, and **(C)** IL-6 levels after 11 weeks of infection. Data represent the mean ± SEM of five mice groups. ** Significant difference in relation to the opposite strain infected by *L. amazonensis*, p ≤ 0.01; ****p ≤ 0.0001.

Considering that *L. amazonensis* infection leads to a differential inflammatory response in BALB/c and C57BL/6 mice, we measured the production of inflammatory cytokines in paws homogenates of infected mice after 11 weeks. C57BL/6 mice presented higher levels of IFN-γ (**Figure 2A**), while BALB/c exhibited higher levels of IL-6 (**Figure 2C**). Eleven weeks after infection, BALB/c and C57BL/6 infected mice did not show different TNF-α levels (**Figure 2B**). For having remained below the technique detection limit, we did not interpret the following values: uninfected mice, IL-2, IL-4, IL-10, and IL-17 values of infected mice, as well as IFN-γ, TNF-α, and IL-6 levels at the infection times from 6 to 240 h.

### C57BL/6 Mice Produce Higher Levels of ${}^{•}O_{2}^{-}$ in the Infection Site Than BALB/c Mice

**Figure 3:**
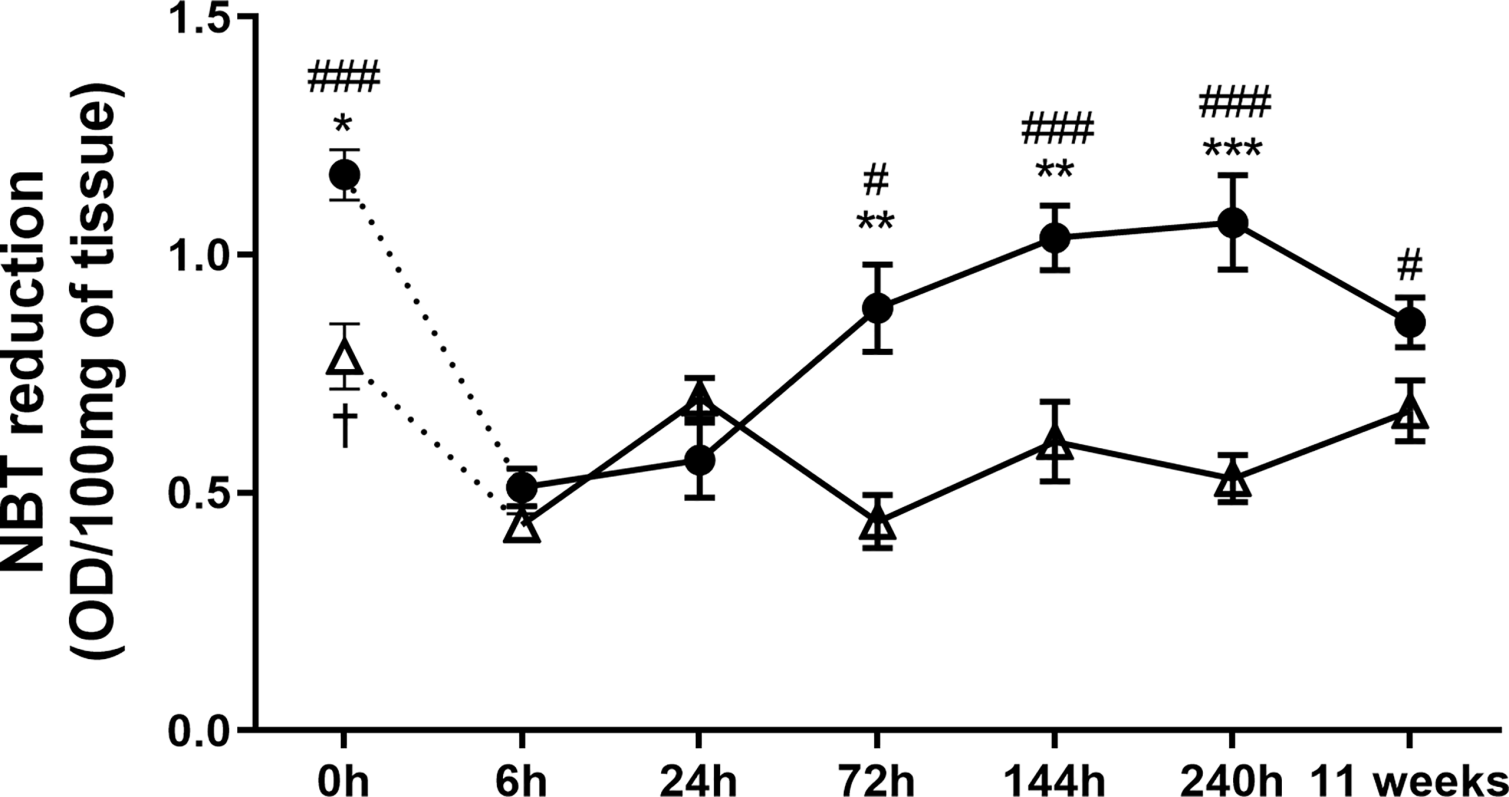
Superoxide anion measured at the paws of BALB/c and C57BL/6 mice infected with *L. amazonensis*. Homogenates of BALB/c and C57BL/6 paws infected with *L. amazonensis* from 6 h to 11 weeks submitted to the NBT assay. Uninfected mice were used as controls (0 h). * Significant difference between mice strains infected by *L. amazonensis*, p ≤ 0.05; **p ≤ 0.01, ***p ≤ 0.001. ^#^Difference in the time points vs. 6 h within C57BL/6 mice, p ≤ 0.05, ^##^p ≤ 0.01, ^###^p ≤ 0.001. †Difference in the time points *vs.* 6 *h* within BALB/c mice, p = 0.016. Data represent the mean ± SEM of six mice groups.

Aiming to understand the mechanisms involved at the early- and late-stage pathogenesis of *L. amazonensis* infection in C57BL/6 and BALB/c mice strains, we measured the ${}^{•}O_{2}^{-}$ production (NBT assay) in paws homogenates. C57BL/6 mice had a higher basal production of ${}^{•}O_{2}^{-}$ (0 h) than BALB/c mice. After infection, these radical levels were drastically reduced in both strains of mice (6h) but increased at later times in the C57BL/6 mice (72 h to 11 weeks). In BALB/c mice, we found that ${}^{•}O_{2}^{-}$ levels remained low for the assessed periods (no statistical difference between 6 h and the later time points) and also presented significantly lower values than C57BL/6 (72 to 240 h) (**Figure 3**).

### BALB/c and C57BL/6 Have Different Macrophage Infiltration and M1 and M2 Markers During *L. amazonensis* Infection

**Figure 4:**
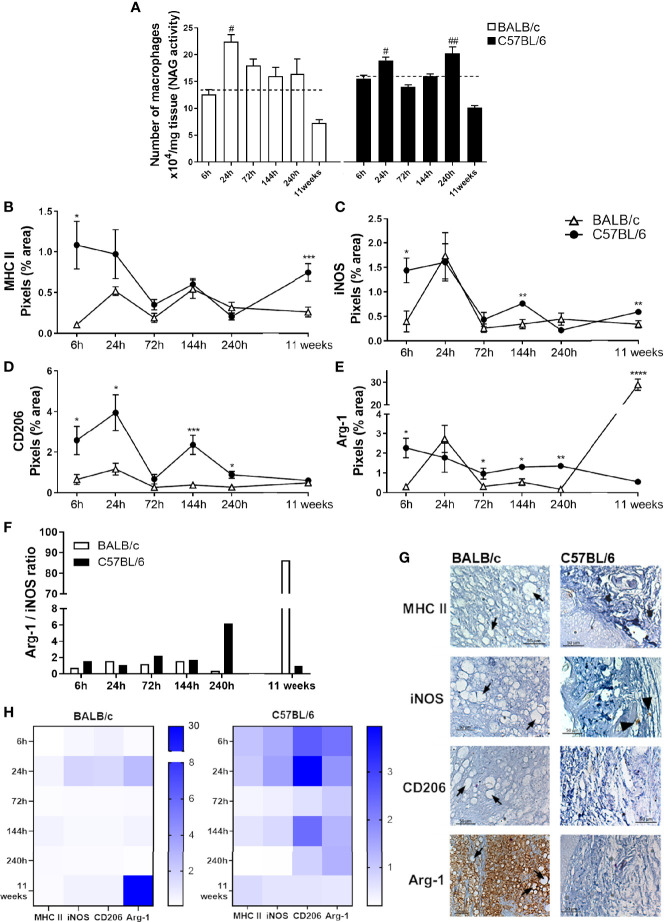
Characterization of local macrophages in infected mice paws. **(A)** Indirect measurement of the macrophage number by NAG activity. **(B)** Immunohistochemical quantification of MHC-II, **(C)** iNOS, **(D)** Arg-1, and **(E)** CD206 staining in the paw of infected mice. ^#^Significant difference in relation to the non-infected mice (dashed lines), p ≤ 0.05; ^##^p ≤ 0.01. *Significant difference between BALB/c and C57BL/6 mice infected with *L. amazonensis*, p ≤ 0.05; **p ≤ 0.01, ***p ≤ 0.001, ****p ≤ 0.0001. **(F)** Arg-1/iNOS ratio. **(G)** Representative figures of immunohistochemical labeling. The positive marking on immunohistochemistry sections corresponds to the brown areas (arrowhead). Arrows indicate vacuolated macrophages filled with parasites. **(H)** Heatmap of MHC-II, iNOS, Arg-1, and CD206 in all evaluated time points. Data from **(A–E)** represent the mean ± SEM (n ≥ 5).

To understand the dynamics of macrophage infiltration at the lesion site, we analyzed the quantity of these cells in the paws throughout the infection. BALB/c mice showed a single peak of macrophage infiltration occurring 24 h post *L. amazonensis* infection; in addition, the amount of local macrophages decreases in sequence (144 h to 11 weeks). In contrast, C57BL/6 showed two peaks of macrophage infiltration, one more initial 24 h after infection, and the later one at 240h p.i. (**Figure 4A**).

To identify the profile of macrophages at the infection site, we performed the immunohistochemistry assay to identify the presence of MHC-II and iNOS labeling related to the M1 profile, as well as CD206 and Arg-1 markers, related to the M2 phenotype. We observed that at early infection (6 h), the C57BL/6 mice show markers from both macrophage subpopulations in relation to BALB/c mice. After 24 h, the time point related to the first macrophage infiltration (**Figure 4A**), C57BL/6 showed high CD206 marking. At 72 h p.i., Arg-1 was higher on C57BL/6 mice paws. After 144 h, C57BL/6 showed higher labeling iNOS, Arg-1, and CD206 than BALB/c mice. At the time point coinciding with the second peak of infiltration, 240 h, C57BL/6 mice presented higher marking for both Arg-1 and CD206 at the infection site. After 11 weeks of infection, this profile was altered, with C57BL/6 presenting more M1 markers, while BALB/c showed significantly higher levels of Arg-1 marking in relation to C57BL/6 (**Figures 4B**–**E**, **G**).

We also found a higher Arg-1/iNOS ratio in C57BL/6 mice than in BALB/c after 240 h of infection. However, after 11 weeks of infection, such balance was reversed, with a substantially higher proportion of marking for Arg-1 in the BALB/c paws compared with C57BL/6 mice (**Figure 4F**).

Furthermore, immunohistochemical images revealed that the intense labeling Arg-1 after 11 weeks of infection coincides with areas with vacuolated macrophages filled with parasites (**Figure 4G**, **Figure S1**). This pattern also appeared in the heatmap (**Figure 4H**), showing a prominent Arg-1 labeling in BALB/c, while the levels found in C57BL/6 were more evenly distributed among the analyzed markers and times.

### C57BL/6 Mice Have More Type III Collagen Deposition at the Infection Site at Late Infection Stages

**Figure 5:**
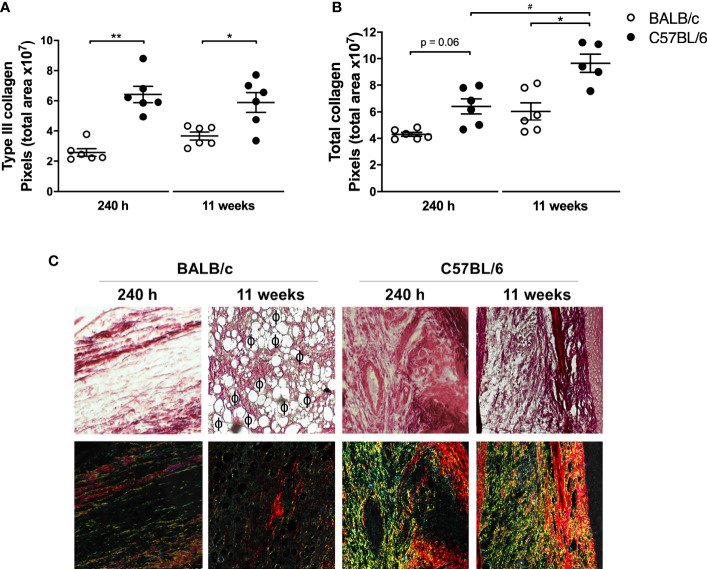
Analysis of collagen in mice paws infected with *L. amazonensis*. Histological sections of the paws of *L. amazonensis*-infected BALB/c and C57BL/6 mice for 240 h or 11 weeks p.i. were stained with Sirius Red to measure **(A)** total collagen and **(B)** type III collagen using the Image-Pro Plus. * Significant difference between the mice strains infected by *L. amazonensis*, p ≤ 0.05; **p ≤ 0.01. ^#^Significant difference of time points in C57BL/6 mice, p ≤ 0.05. **(C)** Representative figures of histological sections of the paws of BALB/c and C57BL/6 mice infected by *L. amazonensis* for 240 h and 11 weeks. ϕ—Indication of some parasitophorous vacuoles. The fibers stained in red correspond to type I collagen and those in yellow/green correspond to deposited type III collagen. Data from **(A, B)** represent the mean ± SEM (n ≥ 5).

Since tissue repair is closely related to the clinical course of leishmaniasis, we identified total collagen and newly deposited collagen (type III) in the paws of infected mice as a readout of tissue repair (Frahs et al., 2018). The results revealed that at 240 h and 11 weeks p.i., C57BL/6 mice had significantly more total and type III collagen than BALB/c (**Figures 5A, B**). In addition, in C57BL/6, the amount of total collagen increased from 240 h to 11 weeks of infection (**Figure 5A**).

**Figure 5C** and **Figure S1** show the presence of vacuolated macrophages containing amastigotes (parasitophorous vacuoles) in BALB/c mice 11 weeks after infection, coinciding with areas of low labeling for type I and type III collagen. In C57BL/6 mice, after 240 h, there were no visible vacuoles in the macrophages and consequently lesser parasites at the infection site. In addition, collagen deposition appeared in the C57BL/6 sections, characterizing a tissue repair process.

### The Macrophages Recruited to the Lesion Site Are Important for the Evolution of Edema and in the Arginase-1 Presence

**Figure 6:**
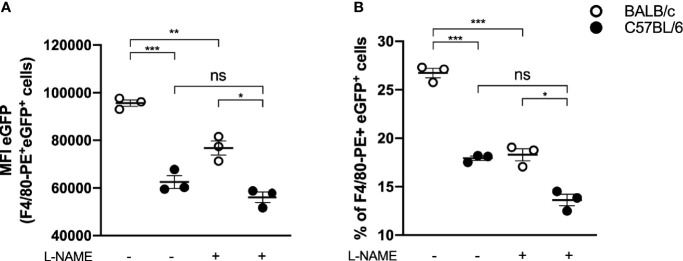
*In vitro* infection of BALB/c and C57BL/6 peritoneal macrophages. Peritoneal macrophages (10^5^) of BALB/c and C57BL/6 mice were infected with 10^6^ e-GPF parasites and evaluated about **(A)** e-GFP median fluorescence intensity (MFI) of macrophages (F4/80^+^ cells) and **(B)** percentage of infected macrophages (% of F4/80^+^ and e-GFP^+^ cells). Data represent the mean ± SEM of three independent experiments performed in duplicate. * Significant difference p ≤ 0.05, **p ≤ 0.01, ***p ≤ 0.001. L-NAME, N(omega)-Nitro-L-arginine methyl ester; NS, nonsignificative.

Considering the differences in the lesion evolution in the studied mouse strains, we investigated the behavior of the respective macrophages against *L. amazonensis* infection *in vitro*. For this purpose, we infected peritoneal macrophages from mice of both strains with eGFP-promastigote forms (1:10) and assessed their ability to eliminate the parasites. We found that macrophages from BALB/c mice had significantly more parasites per macrophage (indicated by eGPF MFI of F4/80^+^ eGFP^+^ cells, **Figure 6A**) and higher percentage of infected macrophages (indicated as percentage of F40/80^+^ and eGFP^+^ cells, **Figure 6B**) after 24 h of infection than C57BL/6. Furthermore, the addition of arginase blocker L-NAME led to an important reduction in infection levels in BALB/c macrophages, while the parasite levels in C57BL/6 mice remained without statistical differences (**Figure 6**).

Interestingly, by adding a blocker, BALB/c macrophage infection levels became similar to C57BL/6 macrophage infection levels in the absence of an inhibitor (17.88 ± 1.87 *vs.* 18.22 ± 2.23, respectively) (**Figure 6B**).

**Figure 7:**
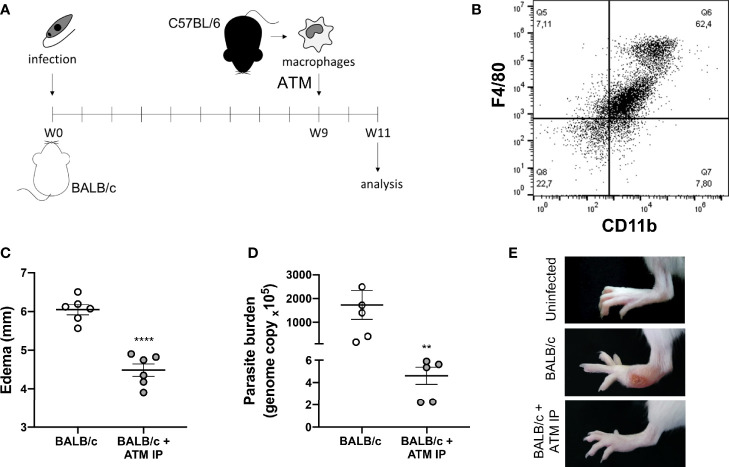
Adoptive transfer of macrophages (ATM). **(A)** Representative scheme of the experimental design: BALB/c mice were infected with *L. amazonensis* and, after 9 weeks, they received peritoneal macrophages isolated from C57BL/6 mice intraperitoneally. **(B)** Dot plot showing the macrophage markers (F4/80^+^/CD11b^+^) in the peritoneal cells from non-infected C57BL/6 mice. **(C)** Paw edema of mice that did not receive adoptive transfer of macrophages (BALB/c) and mice that received intraperitoneal ATM (BALB/c + ATM IP) after 11 weeks of infection. **(D)** Parasite burden and **(E)** representative photographs of mice paws after 11 weeks of infection. Data from **(C, D)** represent the mean ± SEM of five mice groups. ** Significant difference in relation to the group that did not receive ATM, p ≤ 0.01, ****p ≤ 0.0001.

Therefore, for an *in vivo* analysis of the role of recruited macrophages on *L. amazonensis* lesion evolution, we performed the adoptive transfer of macrophages (ATM) from partially resistant mice (C57BL/6) to susceptible ones (BALB/c) after 9 weeks of infection, the time point at which the paw edema starts to significantly differ between the mice strains (**Figures 1A**, **7A**). Firstly, peritoneal cells from C57BL/6 were characterized as predominantly macrophages (F4/80^+^/CD11b^+^) (**Figure 7B**, gate strategy **Figure S2**). Then, we found that mice that received ATM intraperitoneal (ATM IP) presented lower paw edema and more than 250 times less parasite burden (**Figures 7C, D**). Additionally, most ATM animals did not show any ulceration at the infection site, while BALB/c mice presented typical ulcerated lesions (**Figure 7E**).

**Figure 8:**
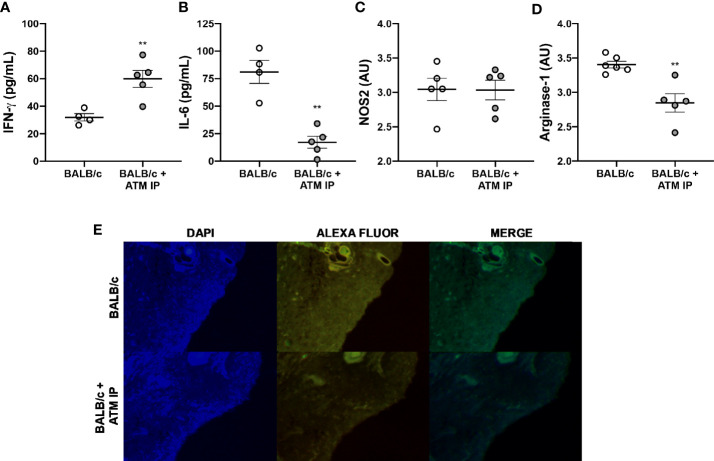
Immunomodulation of ATM mice. Homogenates of *L. amazonensis*-infected BALB/c and BALB/c that received ATM *via* intraperitoneal paws were submitted to **(A)** IFN-γ and **(B)** IL-6 measurement by CBA assay; and **(C)** Arg-1 and **(D)** iNOS measurement by ELISA. Data represent the mean ± SEM of five mice groups. ** Significant difference with the opposite strain infected by *L. amazonensis*, p ≤ 0.01. **(E)** Arginase-1 immunofluorescence study. Cell nucleus was stained with DAPI and arg-1 was stained with Alexa Fluor. ATM, adoptive transfer of macrophages; AU, arbitrary units; IP, intraperitoneal.

Analysis of the local cytokines at the 11th week showed ATM mice with higher IFN-γ levels, while mice that did not receive ATM presented higher IL-6 levels (**Figures 8A, B**). We also verified lower Arg-1 levels in ATM IP mice without significant variation in iNOS compared with BALB/c that did not receive ATM (**Figures 8C, D**). Such reduced Arg-1 levels in ATM animals was further confirmed through immunofluorescence (**Figure 8E**). Similar results emerged by performing ATM *via* the intravenous route (ATM IV), with edema and Arg-1 reduction in the ATM IV group, without significantly different iNOS levels (**Figure S3**).

## Discussion

The pathological mechanisms that lead to cutaneous lesion caused by *L. amazonensis* infection are not yet fully understood. This work aimed to elucidate the role of different molecules and cells by comparing the evolution of *L. amazonensis* infection in mice strains partially resistant and susceptible to infection.

The most studied experimental model of cutaneous leishmaniasis is the *L. major* infection, in which the susceptibility of BALB/c mice has been correlated to the predominance of Th2 immune response, while C57BL/6 mice are resistant to infection, with a predominance of Th1 type response (Sacks and Noben-Trauth, 2002; Scott and Novais, 2016). However, such dichotomy is not identified in the disease induced by other *Leishmania* species, as *L. amazonensis* (Soong, 2012; Scott and Novais, 2016). Our study showed that BALB/c mice had progressive edema from 0 to 11 weeks after *L. amazonensis* infection, with ulceration of the lesion and high local parasitic load 11 weeks p.i. In contrast, C57BL/6 mice presented initial edema that was steadied at the later times analyzed, without ulcerated lesions, in addition to lower parasitic load than BALB/c.

We also found higher production of IL-6 in BALB/c mice in ATM IP mice, which play an important role in the leishmaniasis pathogenesis, triggering the down modulation of the microbicide molecules, and the polarization of macrophages to an M2 phenotype, which are more permissive to *Leishmania* proliferation (Hatzigeorgiou et al., 1993; Hu et al., 2018). Conversely, C57BL/6 mice had a significant increase in IFN-γ levels after 11 weeks of infection, a key cytokine in the activation of inflammatory macrophages (M1), associated with protection in several experimental models of infection (Laskin et al., 2011), including *Leishmania* sp. (Wang et al., 1994; Sacks and Noben-Trauth, 2002).

Moreover, IFN-γ triggers the synthesis of reactive oxygen species (ROS), such as ${}^{•}O_{2}^{-}$ . ROS are produced by M1 macrophages, causing oxidation of lipids, proteins, and nucleic acids, consequently eliminating intracellular parasites (Murray and Wynn, 2011; Jafari et al., 2014). C57BL/6 mice showed higher levels of ${}^{•}O_{2}^{-}$ over the infection, with lower parasitic load, thus explaining a better lesion evolution in this mice strain.

However, an exacerbated pro-oxidant response can be harmful to the host during *Leishmania* sp. infection since the action of ROS is nonspecific and causes damage to the surrounding host cells, consequently leading to inflammation and tissue injury (Morgado et al., 2018; Herb and Schramm, 2021). Therefore, in the presence of pro-oxidant molecules, such as ${}^{•}O_{2}^{-}$ , adaptive homeostasis mechanisms must be activated as a way to protect against tissue damage (Pomatto et al., 2019). Despite producing ${}^{•}O_{2}^{-}$ in a more pronounced manner, mice of the C57BL/6 strain showed higher collagen deposition, suggesting an ability to balance pro- and antioxidant properties, thus explaining the concomitant elimination of the parasite combined with the tissue protection capacity found in C57BL/6 mice (Morgado et al., 2018).

Regarding macrophages, we verified a peak of infiltration in the mice paws of both C57BL/6 and BALB/c mice after 24 h of infection, corroborating the expected kinetics of migration of these cells (Minutti et al., 2017). However, at early infection, there was no clear polarization of macrophages for M1 or M2 phenotype in either mice strain. These data are in accordance with the mixed Th1/Th2 profile induced by *L. amazonensis* infection (Scott and Novais, 2016), as well as with the findings of Ontoria et al. (2018), who found an inconstant distribution of M1 markers throughout the infection by *L. infantum* between 1 and 8 weeks.

The results also showed a second peak of macrophage infiltration in the C57BL/6 mice after 240 h of infection, with higher Arg-1 and CD206 marking than BALB/c. This indicates that macrophages recruited at this point have M2 characteristics, important for tissue remodeling and wound healing (Laskin et al., 2011). The wound healing process is executed and regulated through a signaling network involving numerous growth factors, cytokines, and chemokines. Interestingly, pro- and anti-inflammatory cytokines are important in this process, since the former induces the migration of monocytes to the wound, while the latter is important in the collagen deposition process. Thus, the wound healing is a complex orchestra governed by fine control between responses (Krzyszczyk et al., 2018).

Krzyszczyk et al. (2018) showed that as the tissue begins to recover, the general population of M2 macrophages induces the migration and proliferation of fibroblasts, keratinocytes, and endothelial cells to restore the dermis, epidermis, and vasculature, respectively, by remodeling the injured tissue. In CL wound, such remodeling is marked by the control of inflammation and a higher amount of collagen at the sites from which the parasites have been eliminated (Baldwin et al., 2007; Miranda et al., 2015). Type III collagen is the first deposited in early healing wounds, progressively replaced by type I as scar formation progresses and the tissue remodels (Rangaraj and Harding, 2011). Our data are in agreement with these studies and show that both collagen type levels were higher in C57BL/6 mice than in BALB/c mice, confirming the better lesion evolution in this mice strain. Additionally, collagen deposition was higher in areas with low parasitic load and tissue reorganization in the C57BL/6 mice. We also found a reduction in ${}^{•}O_{2}^{-}$ in C57BL/6 after 11 weeks of infection, which is in accordance with the higher levels of collagen, since a new tissue formation stage is characterized by lower ROS levels (Xu et al., 2017).

Despite an infiltration of M2 macrophages in 240 h in C57BL/6 mice, in the later period studied, 11 weeks, a shift for M1 macrophages occurred, coinciding with higher levels of IFN-γ, with less parasitic load and no apparent lesion. In contrast, BALB/c mice had worse lesion evolution and a marked increase in Arg-1 (a M2 macrophage marker) in the later period analyzed (Muraille et al., 2014). Arginase-1 plays a fundamental role in the survival and multiplication of intracellular amastigotes of *Leishmania* sp., bypassing the amino acid L-arginine for the preferential synthesis of polyamines, fundamental for the parasite’s nutrition (Muxel et al., 2017).

*In vitro* studies showed that *L. amazonensis-*infected BALB/c macrophages present increased L-arginine uptake and expression of arginase and arginase-related genes (Muxel et al., 2017; Aoki et al., 2019). Our data agree with these studies and show that *in vitro* BALB/c macrophages have a lower parasite clearance capacity than C57BL/6 macrophages; however, when arginase was blocked, parasite clearance by BALB/c macrophages increases substantially, reaching levels similar to those of C57BL/6. It has been reported that arginase activity is inhibited by arginine analog L-NAME, both *in vitro* and *in vivo*; however, its function as an iNOS inhibitor has also been proved (Reisser et al., 2008). Since NO is important for eliminating *L. amazonensis*, reducing its levels would generate a larger number of intracellular parasites (Scott and Novais, 2016); therefore, we believe that in our experiments, L-NAME acted mainly by inhibiting arginase and not iNOS.

The role of Arg-1 from macrophages on CL lesions was further demonstrated through the adoptive transfer of macrophages from C57BL/6 to BALB/c mice, which revealed that mice receiving the cells presented better disease outcome, with similar features to those found in partially resistant mice. The recruited macrophages have been described as important weapons in *Leishmania* control, since the tissue resident macrophages present oxidative deficiency in relation to the recruited ones (Pessenda and Santana da Silva, 2020).

An important role for arginase in the susceptibility of mice to *L. major* infection had been described (Kropf et al., 2005), as well its action in *L. amazonensis* infection *in vitro*. Interestingly, skin biopsies and plasma from CL patients also present high levels of Arg-1 [reviewed in (Muxel et al., 2017)]. However, until now, little was known about Arg-1 in *L. amazonensis* infection *in vivo*. We showed that the participation of this enzyme in the susceptibility of animals is particularly important at later infection.

Peritoneal macrophages are a heterogeneous population of M0, M1, and M2 cells, with the capacity to differentiate or redifferentiate into both in M1 and M2 phenotypes (Zhao et al., 2017). Furthermore, these cells are recruited to other tissue under infection or inflammation conditions (Cassado et al., 2015). In this context, we performed the adoptive transfer of macrophages from C57BL/6 to BALB/c mice, whose results suggest a great importance of the recruited macrophages in controlling susceptibility to *L. amazonensis*. The transfer of macrophages from partially resistant mice (C57BL/6) to a susceptible mouse (BALB/c) modulates the pro-*Leishmania* environment to an anti-leishmania state, with high IFN-γ and low Arg-1 levels, less parasite burden and edema, and better disease outcome.

## Conclusion

Taking together, the results suggest that a balance between a pro-inflammatory and microbicide M1-related and a tissue restorative M2-related response seems to provide the utmost benefit for the host. We also demonstrated that higher levels of Arg-1 at late infection periods modulate the disease outcome in susceptible mice, but the transference of macrophages from partially resistant mice (C57BL/6) restores the host protection and disease control. These results expand our understanding on the protective immunity against *L. amazonensis*, providing new insights on potential interventions to treat or prevent the disease.

## Data Availability Statement

The raw data supporting the conclusions of this article will be made available by the authors, without undue reservation.

## Ethics Statement

The animal study was reviewed and approved by the Ethics Committee for Animal Experimentation of the State University of Londrina.

## Author Contributions

FT-P: Conceptualization, data curation, formal analysis, investigation, writing—original draft, and project administration. MM-S: Methodology, data curation, formal analysis, investigation, writing—original draft, and supervision. TS, BB, MG, and VC: Methodology, data curation, formal analysis, investigation, and writing—review and editing. VC, AR, MD, LS-F and WVJ: Methodology, data curation, and formal analysis. IC and IC-C: Project administration, supervision, writing—review and editing, and resources. CP: Methodology, data curation, formal analysis, and writing—review and editing. JB: Conceptualization, supervision, validation, writing—review and editing, and resources. WP: Conceptualization, project administration, supervision, writing—review and editing, and resources. All authors contributed to the article and approved the submitted version.

## Funding

This study was partly financed by the Coordenação de Aperfeiçoamento de Pessoal de Nível Superior—Brazil (CAPES) [Finance Code 001]. WP [301594/2018-0], IC-C [303233/2017-0], and JB [312671/2020-2] are CNPq fellows.

## Conflict of Interest

The authors declare that the research was conducted in the absence of any commercial or financial relationships that could be construed as a potential conflict of interest.

## Publisher’s Note

All claims expressed in this article are solely those of the authors and do not necessarily represent those of their affiliated organizations, or those of the publisher, the editors and the reviewers. Any product that may be evaluated in this article, or claim that may be made by its manufacturer, is not guaranteed or endorsed by the publisher.
