# Corrigendum: Murine Susceptibility to *Leishmania amazonensis* Infection Is Influenced by Arginase-1 and Macrophages at the Lesion Site

**DOI:** 10.3389/fcimb.2021.804809

**Published:** 2022-02-24

**Authors:** Fernanda Tomiotto-Pellissier, Milena Menegazzo Miranda-Sapla, Taylon Felipe Silva, Bruna Taciane da Silva Bortoleti, Manoela Daiele Gonçalves, Virginia Márcia Concato, Ana Carolina Jacob Rodrigues, Mariana Barbosa Detoni, Larissa Staurengo-Ferrari, Waldiceu Aparecido Verri, Idessania Nazareth Costa, Carolina Panis, Ivete Conchon-Costa, Juliano Bordignon, Wander Rogério Pavanelli

**Affiliations:** ^1^Biosciences and Biotechnology Graduate Program, Carlos Chagas Institute (ICC), Fiocruz, Curitiba, Brazil; ^2^Laboratory of Immunoparasitology of Neglected Diseases and Cancer (LIDNC), Department of Pathological Sciences, State University of Londrina, Londrina, Brazil; ^3^Laboratory of Biotransformation and Phytochemistry, Department of Chemistry, State University of Londrina, Universitary Hospital, Londrina, Brazil; ^4^Laboratory of Pain, Inflammation, Neuropathy and Cancer, Department of Pathology, Center for Biological Sciences, State University of Londrina, Londrina, Brazil; ^5^Laboratory of Tumor Biology, State University of Western Parana´ (UNIOESTE), Francisco Beltrão, Brazil; ^6^Laboratory of Molecular Virology, Carlos Chagas Institute (ICC), Fiocruz, Curitiba, Brazil

**Keywords:** *Leishmaniasis*, M1, M2, IFN-γ, wound healing, collagen, iNOS

## Publisher’s Note

All claims expressed in this article are solely those of the authors and do not necessarily represent those of their affiliated organizations, or those of the publisher, the editors and the reviewers. Any product that may be evaluated in this article, or claim that may be made by its manufacturer, is not guaranteed or endorsed by the publisher.

## Addition of an Author

**Larissa Staurengo-Ferrari and Waldiceu Aparecido Verri Jr** were not included as authors in the published article. The corrected Author Contributions Statement appears below.

The authors apologize for this error and state that this does not change the scientific conclusions of the article in any way. The original article has been updated.

**FT-P: Conceptualization, data curation, formal analysis, investigation, writing—original draft, and project administration. MM-S: Methodology, data curation, formal analysis, investigation, writing—original draft, and supervision. TS, BB, MG, and VC: Methodology, data curation, formal analysis, investigation, and writing—review and editing. VC, AR, MD, LS-F and WVJ: Methodology, data curation, and formal analysis. IC and IC-C: Project administration, supervision, writing—review and editing, and resources. CP: Methodology, data curation, formal analysis, and writing—review and editing. JB: Conceptualization, supervision, validation, writing—review and editing, and resources. WP: Conceptualization, project administration, supervision, writing—review and editing, and resources. All authors contributed to the article and approved the submitted version**.

