# Supplementary material for: Murine Susceptibility to Leishmania amazonensis Infection Is Influenced by Arginase-1 and Macrophages at the Lesion Site
Source: Front Cell Infect Microbiol. 2021 Oct 1;11:687633. doi: 10.3389/fcimb.2021.687633 (PMC8517480; doi:10.3389/fcimb.2021.687633)
Supplement: Supplementary file 1 [file DataSheet_1.docx]

**SUPPLEMENTARY FIGURES**

**
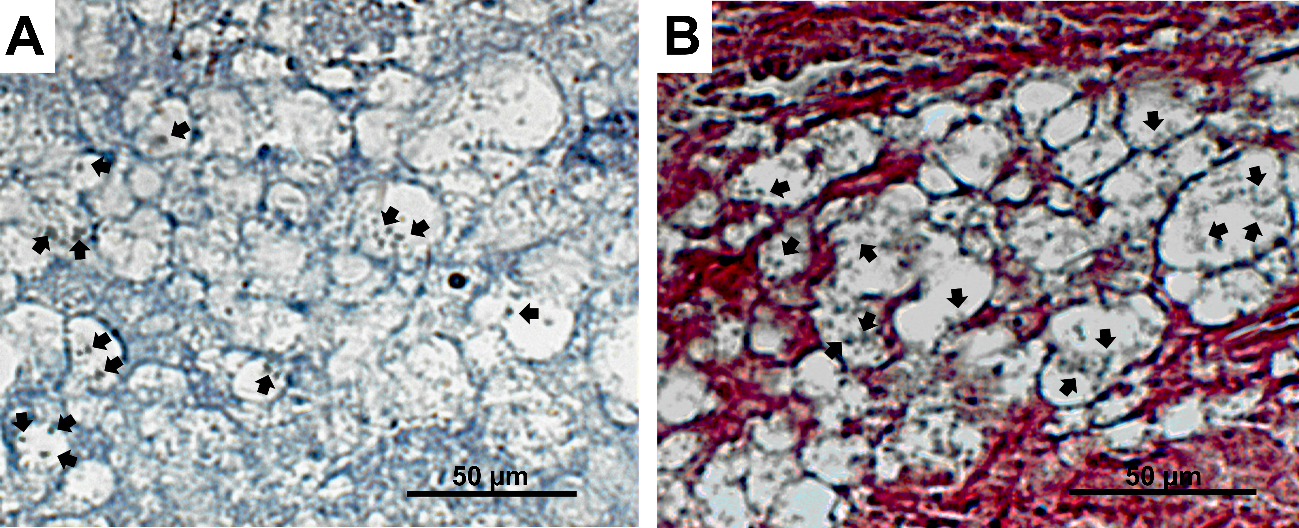
**

**Figure S1 – Figure S1 – Parasitophorous vacuoles containing amastigotes in paws of BALB/c mice infected with *L. amazonensis* for 11 weeks.** (A) Lesion areas of cuts stained with hematoxylin. (B) Lesion areas of cuts stained with Sirus Red. Both images show macrophage vacuoles filled with parasites. The arrows indicate *L. amazonensis* amastigote forms inside parasitophorous vacuoles. Scale bar indicates 50 µm.

**
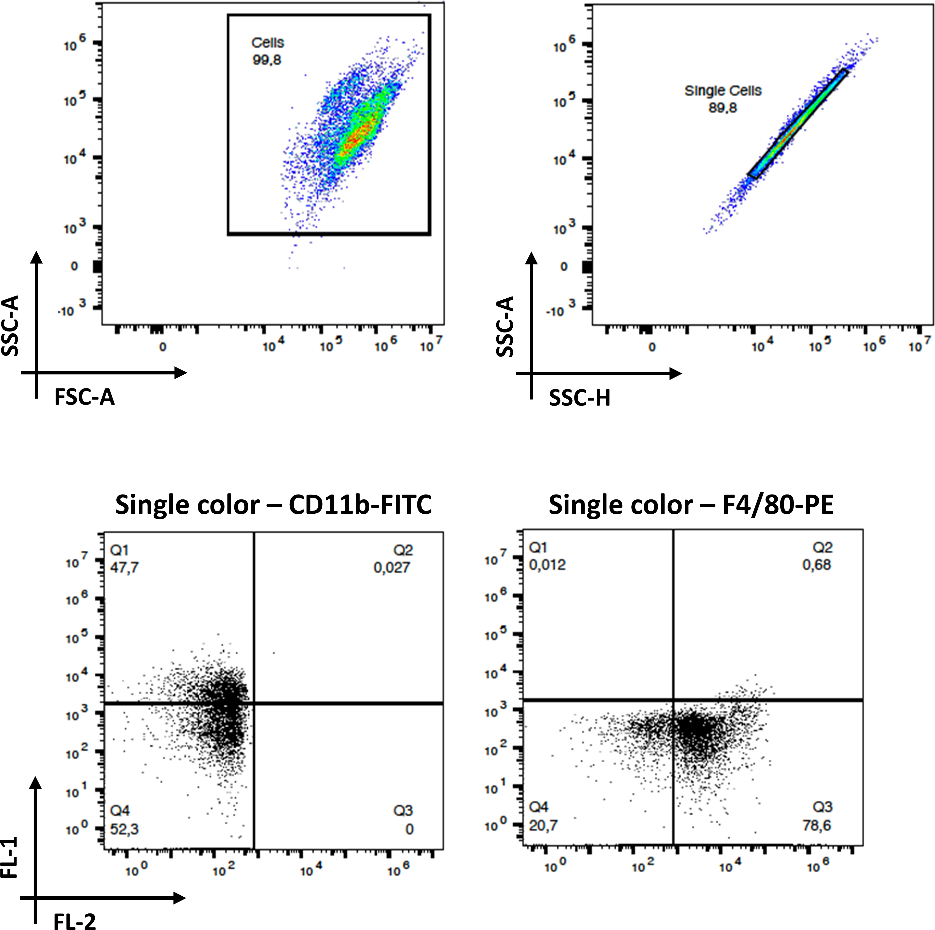
**

**Figure S2 – Flow cytometry gating strategy for macrophages.** (A) Cells were first identified by a forward scatter (FSC) and side scatter (SSC) gate. (B) Gating strategy for identifying singlets (single cells). (C) Single‐color compensation controls for each fluorophore. Flow cytometry plots are shown in logarithmic format.

**
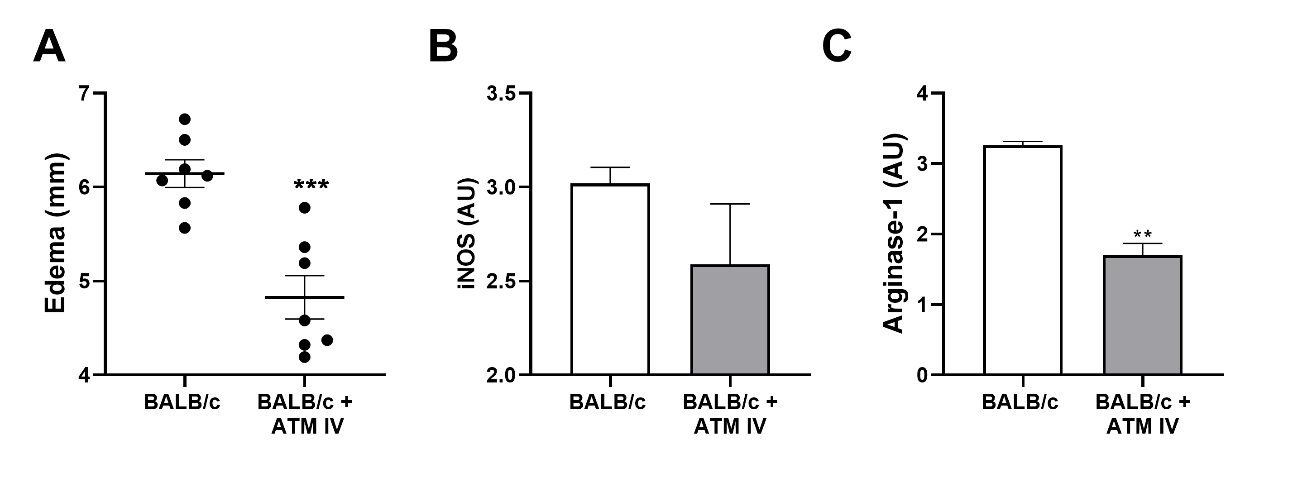
**

**Figure S3 – Analysis of intravenous ATM.** (A) Paw edema of mice that did not receive the adoptive transfer of macrophages (BALB/c) and mice that received intravenous ATM (BALB/c + ATM IV) after 11 weeks of infection. Paws homogenates were submitted to the mensuration of (B) iNOS levels and (C) Arg-1 levels. Data represent the mean ± SEM of 5 mice group. ** Significant difference with the group that did not receive ATM p ≤ 0.01, *** p ≤ 0.001. ATM – adoptive transfer of macrophages. AU – arbitrary units. IV – intravenous.
